# Utilization of images and three-dimensional custom-made nostril retainer fabricate for patients with cleft lip and cleft lip nose deformities at Siriraj Hospital: preliminary phase

**DOI:** 10.1038/s41598-023-46327-1

**Published:** 2023-11-04

**Authors:** Irin Chaikangwan, Nutcha Yodrabum, Worapan Kusakunniran, Rachata Tachavijijaru, Chongdee Aojanepong

**Affiliations:** 1 Division of Plastic and Reconstructive Surgery, Department of Surgery, Faculty of Medicine Siriraj Hospital, Mahidol University, Bangkok, 10700 Thailand; 2 Faculty of Information and Communication Technology, Mahidol University, Nakhon Pathom, Thailand

**Keywords:** Anatomy, Medical research, Preclinical research

## Abstract

A prospective study utilizing image analysis to assess nostril openings in post-operative patients with cleft lip and cleft lip nose deformities. This preliminary study seeks to employ two-dimensional (2D) images to fabricate a custom-made nostril retainer. This study was performed at Faculty of Medicine, Siriraj Hospital, Mahidol University, Thailand. This study included 30 healthy volunteers and 15 patients with cleft lip and cleft lip nose deformities. The nostril opening width and height for all participants were measured, and photographs were taken. An image analysis application was used to fabricate a three-dimensional (3D) custom-made nostril retainer. The mean differences between the direct measurements of the nostril aperture and the measurements obtained through the program did not exceed 2 mm in terms of nostril height, width, or columella. Two-dimensional photographs can be used to create a custom-made, three-dimensional nostril retainer. This retainer allows post-operative patients to maintain their nares without needing to visit the hospital, thereby reducing the cost of care.

## Introduction

Cleft lip and cleft lip nose deformities rank among the most prevalent congenital anomalies affecting children globally. These anomalies significantly impact children's quality of life, functionality, and appearance, leading to challenges in feeding, speech, and social assimilation. Comprehensive multidisciplinary care, stretching from birth to adulthood, becomes crucial. Although surgical interventions for cleft lip and cleft lip nose deformities have seen continuous evolution, aiming at enhanced functional and aesthetic results, the postoperative phase still poses challenges, including potential complications, less-than-optimal outcomes, and the possibility of secondary surgeries.

Symmetry in surgical procedures is pivotal, especially when addressing the nostril opening—a readily noticeable feature. Post-surgical maintenance and prevention of nasal cavity collapse are essential. Given that Asian noses often have underdeveloped alar cartilage, thin alar cartilage, and thick skin, the surgically corrected nose is susceptible to relapse. The nostril retainer proves beneficial in maintaining the results of surgical repair for cleft-lip nose deformities^1,2^.

Extensive research has introduced diverse shapes and materials for nostril retainers, all designed to maintain post-surgical corrections. Common materials for these retainers include acrylic and silicone. In 1994, Dhanraj et al.^3^ employed suction rubber tubes to prevent post-operative nostril stenosis. Grayson et al.^4^ introduced various shapes and materials for nasoalveolar molding, primarily targeting cleft lip and palate anomalies before surgery. Yeow et al.^5^ found similar results when comparing a post-operative nostril retainer cohort with a control group. Yuzuriha et al.^6^ developed a unilateral nostril retainer, designed to be discreet yet able to firmly support the corrected nasal shape of cleft lip surgery patients. Utilizing nostril retainers for cleft lip and cleft lip nose deformities management has demonstrably contributed to superior surgical outcomes, ensuring the maintenance of the improved nasal form and reducing postoperative complications^7^.

Thailand's market offers limited nostril retainer varieties for cleft lip and palate patients. In our facility, we provide two primary types of nostril retainers. The first is a commercial silicone nostril retainer produced by Koken Co. Ltd., Tokyo, Japan. Unfortunately, its cost can be prohibitive for some patients, as medical insurance does not cover it. An alternative is nostril retainers fashioned from more affordable medical supplies, such as nasogastric tubes or infant-sized endotracheal tubes. Due to financial constraints, certain patients resort to this less ideal solution.

Further challenges arise even with commercial products. For patients with nostrils of differing sizes, standard symmetrical retainers might suit one nostril but not the other. This mismatch also occurs when surgeons need an asymmetrical retainer for excessive anterior nasal recess correction^6^. Custom nostril retainers can effectively address these issues.

During the COVID-19 pandemic, another challenge emerged: patients found it difficult to attend follow-up appointments, relying instead on telemedicine. For those infected with the virus, direct contact with physicians became impossible, complicating the process of accurately measuring for nostril retainer selection.

The integration of three-dimensional (3D) technology with medical imaging has transformed various healthcare facets, especially surgical planning and implementation. Recent research employed sophisticated image analysis methods, indicating that 3D facial structure alterations can predict surgical results, regardless of a surgeon's training and experience^8,9^. Kimura et al.^10^ harnessed 3D facial data from twenty-two unilateral cleft lip and palate patients to analyze facial surface asymmetry, offering insights for enhancing muscle reconstruction in cleft lip repairs.

In this context, custom-made nostril retainers designed using 3D technology and medical images have the potential to offer more accurate and patient-specific solutions.

Among these limitations in sources of nostril retainer, those manufactured in various shapes and materials are often neither affordable nor individualized^11,12^. Our study aims to fabricate individualized nostril retainers using multiple perspectives from patients’ two-dimensional (2D) photography and image analysis to design a 3D nostril retainer. This study's objective is to achieve individualized fit for the fabricated nostril retainer, addressing patients’ deformities and offering cost savings compared to commercial retainers.

## Methods

### Study subjects

In the preliminary phase of this investigation conducted at Siriraj Hospital, a cohort of thirty healthy volunteers and fifteen post-cheiloplasty patients were meticulously selected for study. It was imperative to omit from the study those volunteers who had a documented history of rhinoplasty or any facial traumas. Concurrently, any patient presenting with a known history of syndromic anomalies or a documented hypersensitivity to acrylic resin was precluded from participation.

All protocols and methodologies employed in this study strictly adhered to the ethical guidelines delineated in the Declaration of Helsinki. Institutional approval was secured from Siriraj Hospital’s Institutional Review Board, as evident from protocol number 174/2564 (IRB4) (COA no. Si 536/2021). It is paramount to emphasize that all interventions and data collections were executed in congruence with prevailing regulations.

Acquisition of informed consent was paramount. All data pertaining to the patients were meticulously procured post obtaining an unequivocal informed assent from the participants, and in cases involving minors, their legal guardians. Moreover, a written consent was acquired from the participants and their guardians, should it be necessary, bestowing permission to disseminate their photographs for academic purposes.

A salient feature of this research was the conceptualization and realization of the novel Automatic reshaping 3D nostril retainer application. This avant-garde endeavor was the culmination of a synergistic collaboration between the Division of Plastic and Reconstructive Surgery at the Faculty of Medicine, Siriraj, Mahidol University, and the Faculty of Information and Communication Technology at Mahidol University. This application underscores the prodigious potential of three-dimensional technology in advancing the domain of medical interventions and diagnostics.

### Measurement of nostril dimensions

**Figure 1:**
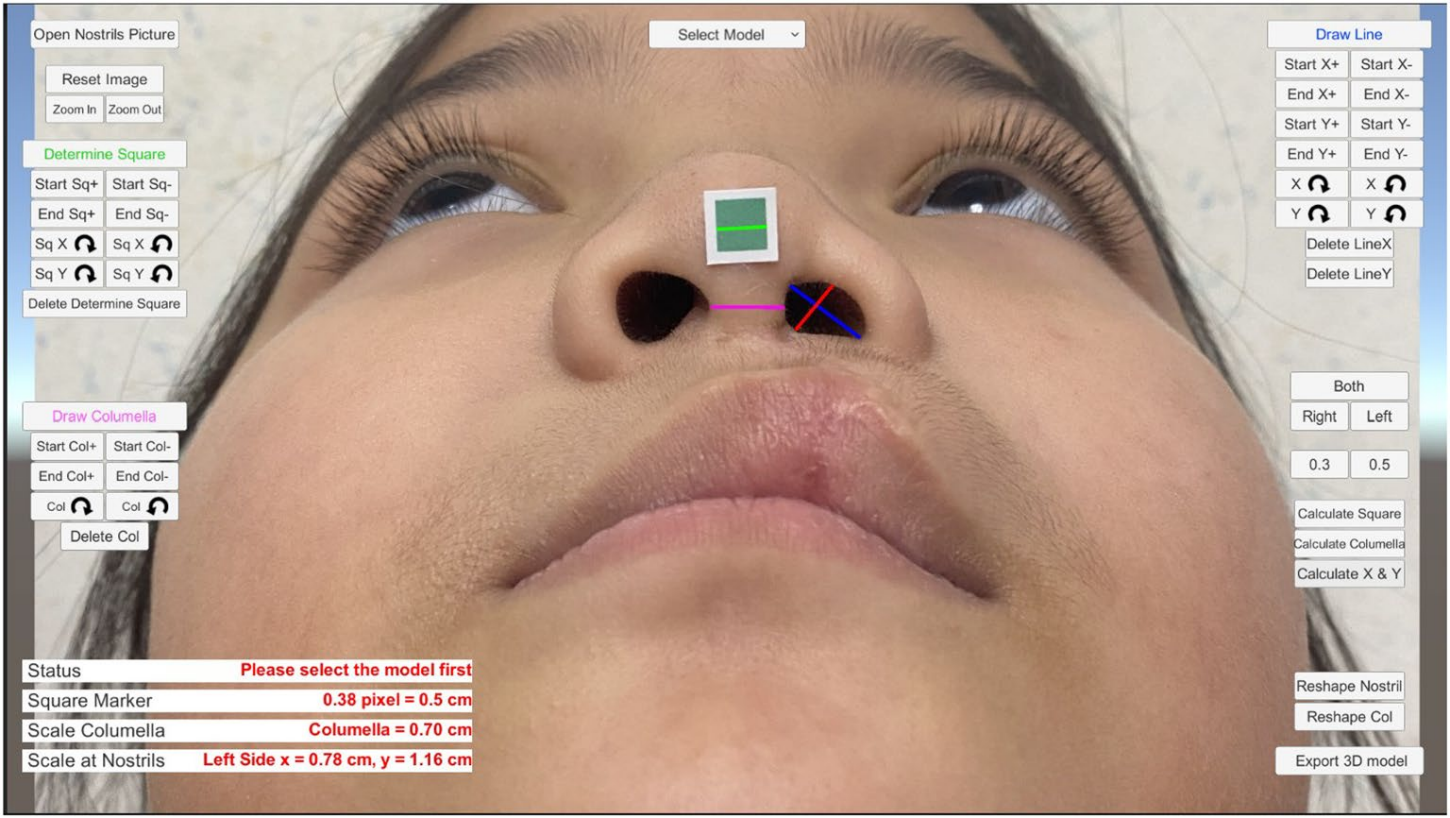
Demonstrate the interface of the program for building a customized three-dimensional nostril retainer following the importation of a two-dimensional basilar view image and the marking of all lines. In this instance, the program scale is equivalent to 0.5 cm and the green line represents the calibrator of the real world. The blue line indicates the height of the nose opening, or the Y axis, for our software. The width of the nose opening, or X axis, is represented by the red line. The pink line indicates columella width.

A meticulous anatomical assessment of the nares was undertaken for both the cohort of healthy volunteers and the patients afflicted with cleft lip anomalies. The parameters under scrutiny included the width, height, and columellar dimensions of the right and left nostril orifices. In the context of this study, the maximum dimensions seen in the basilar view, both in terms of width and height of the nostril aperture, have been illustrated in Fig. 1.

A dual methodology was adopted for this analytical endeavor. Firstly, a direct measurement technique was employed, utilizing digital calipers (TIGA^®^, 150 mm × 0.05 mm/6″ × 1/128in) boasting a remarkable precision of 0.01 mm. Complementing this, a program-centric measurement strategy was also harnessed, leveraging specialized computer-aided software tailor-made for this particular analysis. While the former strategy was contingent upon the physical dimensions procured directly from the participants, the latter was dependent on the digital representations or scanned imagery of the participants’ nares to extrapolate the requisite dimensions.

To mitigate any biases and to assure consistency and accuracy, a singular investigator, proficiently trained, was entrusted with the responsibility of all the measurements. The participants were instructed to assume an erect seated posture with their cranium steadfastly aligned with the Frankfort horizontal plane. To further bolster the precision, each specific measurement was replicated thrice, and the mean value derived from these was chosen for the ensuing analytical processes. One of the pivotal objectives of this research was to discern the variations in the dimensions of the nares, both inter-gender and between the healthy and cleft lip-affected cohorts. Additionally, an evaluation of the congruence between the direct and software-assisted measurement techniques was also undertaken.

### 2D basilar view photography

Before initiating the photographic procedure, a green calibration marker, either measuring 0.3 × 0.3 cm or 0.5 × 0.5 cm, will be strategically placed at the nasal tip of the patients. This acts as a reference for size calibration in the ensuing analysis. The next step involves capturing the basilar view images using a high-definition smartphone camera, specifically the iPhone 11 Pro Max, which boasts a triple lens camera system with a resolution of 12 MP. Upon successful capture, these images are promptly uploaded to the dedicated application for further examination and analysis. A representative image of this process can be viewed in Supplementary Fig. 1.

### 3D model creation and customization of nostril retainer using 2D photography

Utilizing the advanced capabilities of Blender software (version 2.93.1), an archetypal 3D model depicting a symmetrical nostril retainer was generated as a foundational template. Parameters extracted from 2D photographic representations play a pivotal role in tailoring this pre-existing 3D model.

Upon successful image upload, personalized nostril opening adjustments are executed using the Unity software (version 2019.4.12f1). The software facilitates the delineation of an x-coordinate line representing nostril width and a y-coordinate line indicative of nostril height. Importantly, these coordinates are amenable to edits or removal to ensure a precise representation of the patient's nostril anatomy. Especially in cases of unilateral deformities, the capability to fine-tune each nostril separately becomes imperative. The software's flexibility extends to patients with post-operative overcorrection, allowing for meticulous adjustments to craft a nostril retainer tailored to the individual.

The application discerns the pixel count from the line drawn on the green square, subsequently calibrating this to either 0.3 or 0.5 cm, which mirrors the real-world dimensions of the green calibration marker. Essential metrics, such as the dimensions of the nostril openings and columellar width, are derived from these calibrated values. Following this, the software computes a scaling factor, effectively rescaling the default models, culminating in the formation of a patient-specific model.

**Figure 2:**
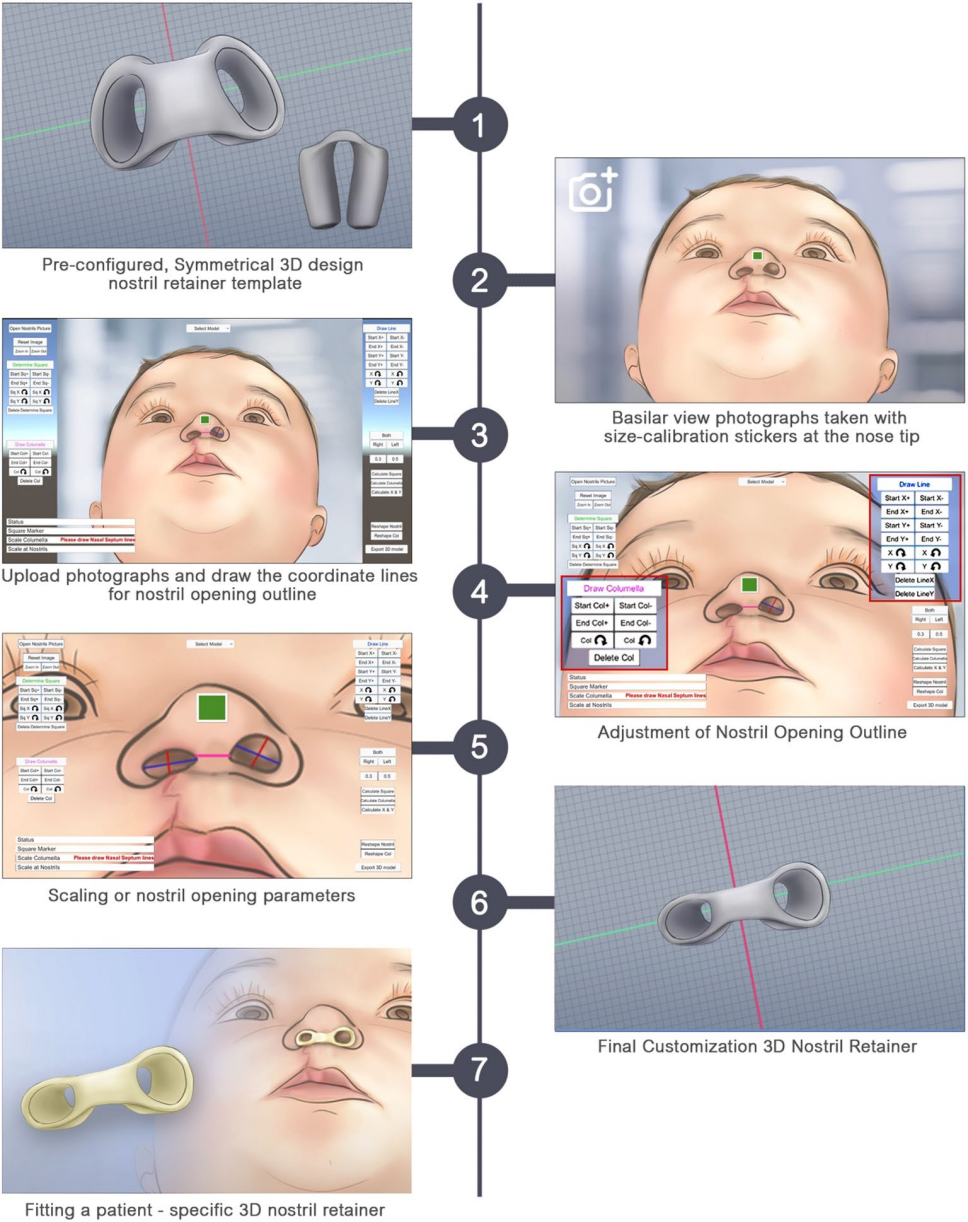
Workflow for custom-made 3D nostril retainer fabrication.

Reverting to Blender, this patient-specific design is translated into a tangible 3D nostril retainer. After meticulous design adjustments, the retainer is saved as an STL file, a universally accepted 3D model format, ensuring seamless interoperability with 3D printers and related software. The final fabrication phase commences with the STL file's transmission to a 3D printer. Here, layer-by-layer deposition of acrylic resin meticulously replicates the digital design, yielding a structurally robust, patient-specific nostril retainer. A comprehensive overview of this intricate application workflow can be viewed in Fig. 2.

### Data analysis and evaluation

To gauge the precision of the 3D nostril retainers, measurements from healthy subjects and cleft lip patients were analyzed using SPSS software (version 25.0, IBM Corp., Armonk, NY, USA). Direct measurements were compared with program-derived ones.

Statistical tests like the Paired t-test or the Wilcoxon signed-rank test were utilized, depending on the data distribution. Comparisons were made between groups, and a p-value under 0.05 denoted statistical significance.

The Intraclass Correlation Coefficient (ICC) was employed to determine reliability. ICC values below 0.5 signified poor reliability, between 0.5 and 0.75 indicated moderate, from 0.75 to 0.9 was good, and over 0.9 was excellent.

Bland–Altman plots were created to visualize the agreement between the two measurement methods. These plots displayed the mean difference between the methods against their average. Limits of agreement were set as the mean difference plus or minus 1.96 times the standard deviation of the difference.

A cost analysis compared the production expenses of the custom-made 3D retainers to standard commercial retainers. Finally, feedback on the retainer's fit, comfort, and user-friendliness was collected from patients and medical professionals.

## Results

### Healthy volunteer

In a study involving 30 Thai volunteers, predominantly female (76.7%), with an average age of 40.3 ± 9.6 years, the anatomy of the nares was meticulously measured. Both right and left nostril openings were assessed in terms of width, height, and columella. Notably, no significant differences were found between the right and left nostrils in terms of width and height.

**Figure 3:**
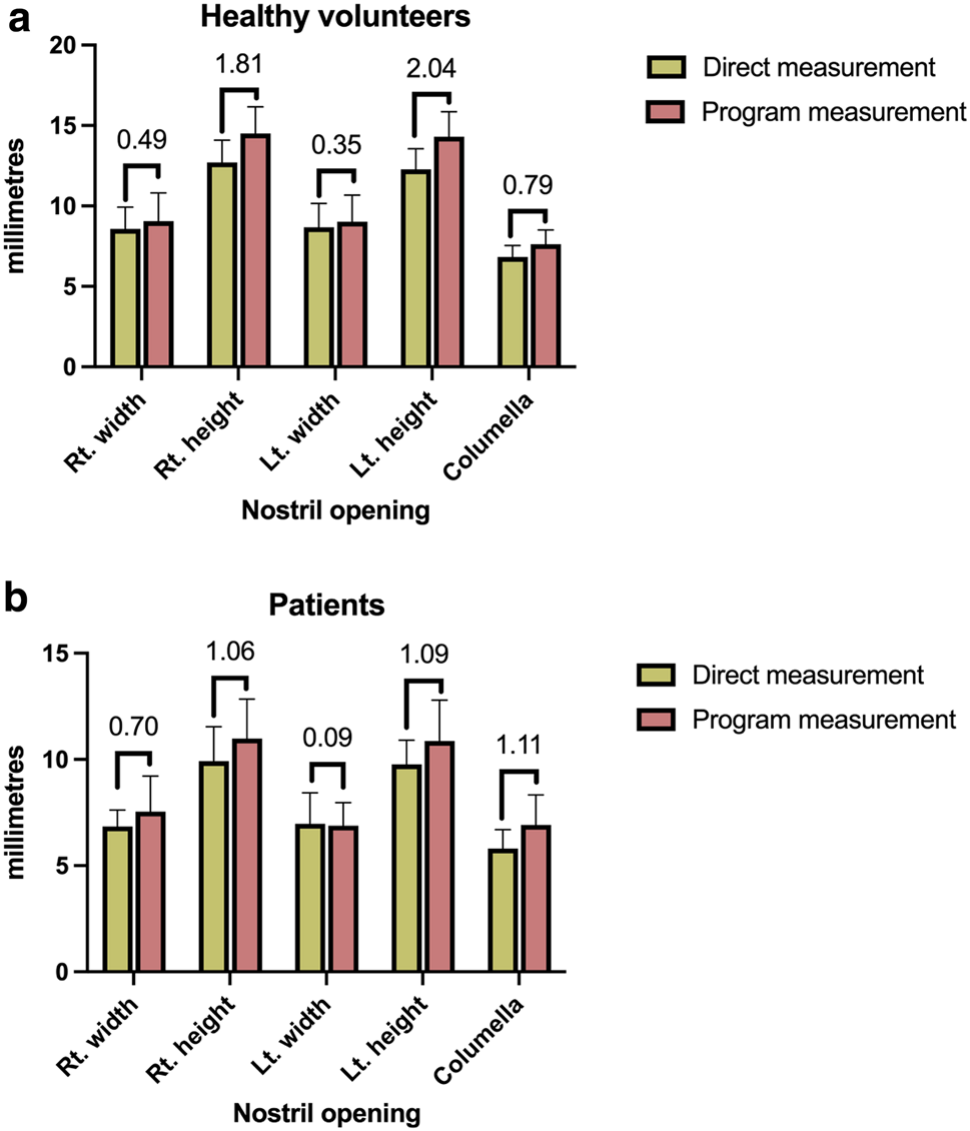
(**A**) Difference between direct nasal aperture measurement with vernier calipers and program measurement in healthy volunteers. (**B)** Difference between direct nasal aperture measurement with vernier calipers and program measurement in cleft patients.

However, a distinction in nostril height between males (13.2 mm) and females (12.3 mm) was observed. The average widths were 8.4 mm for males and 8.7 mm for females (Table 1). Comparing direct to program-based measurements, discrepancies were 0.5 mm and 0.4 mm for the right and left nostril widths, respectively, with nostril height differing by 1.8 mm on the right and 2.0 mm on the left. The columella showed a mean difference of 0.8 mm (Fig. 3a).

**Table 1:** Gender-based distinction in nostril opening measurement of healthy volunteer.

Parameter	Female (n = 23)	Male (n = 7)	Total (n = 30)	p-value						
Age (year)	42.8 ± 9.2	31.9 ± 4.9	40.3 ± 9.6	0.01						

Parameter	Female (n = 23)			Male (n = 7)			Total (n = 30)			p-value
	Right	Left	Both	Right	Left	Both	Right	Left	Both	
Measurement of nostril opening										
Height (mm)	12.5 ± 1.4*	12.0 ± 1.3*	12.3 ± 1.4**	13.3 ± 1.0	13.0 ± 1.0	13.2 ± 1.0**	12.7 ± 1.4	12.3 ± 1.3	12.5 ± 1.3	0.216
Width (mm)	8.7 ± 1.4	8.7 ± 1.5	8.7 ± 1.4	8.3 ± 1.3	8.4 ± 1.4	8.4 ± 1.3	8.6 ± 1.4	8.7 ± 1.5	8.6 ± 1.4	0.793
Columella (mm)	6.7 ± 0.6			7.2 ± 0.9			6.8 ± 0.7			0.160

*Significant difference between side of nostril opening.

**Significant difference between gender.

**Figure 4:**
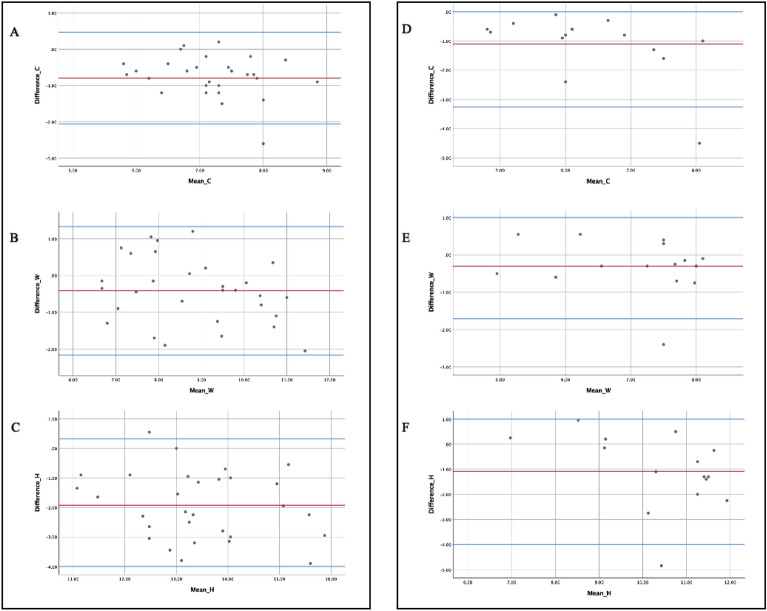
Bland–Almont plot for direct and program measurement agreement. (**A**,**B**) Among healthy individuals, (**A**) represents columella agreement. (**B**) Represents the nostril's width, whereas (**C**) represents its height. Cleft patients with (**D**–**F**) showed agreement. (**D**) denote agreement of columella. (**E**) Represents the nostril's width, whereas (**F**) represents its height.

The Bland–Altman plot confirmed the agreement between the two measurement techniques, with 95% of measurements falling within two standard deviations of the mean (Fig. 4a–c). The intraclass correlation coefficients indicated moderate to excellent reliability across metrics (Table 2).

**Table 2:** Intra- and inter-rater reliabilities of direct and program-based volunteers’ nostril opening measurement.

Parameters	Intra-rater reliability ICC (95% CI)		Inter-rater reliability ICC (95% CI)	
	Direct measurement	Program measurement	Direct measurement	Program measurement
Width of right nostril opening	0.968 (0.917–0.986)	0.992 (0.986–0.996)	0.918 (0.829–0.961)	0.963 (0.932–0.981)
Height of right nostril opening	0.981 (0.956–0.990)	0.992 (0.986–0.996)	0.813 (0.659–0.905)	0.963 (0.923–0.982)
With of left nostril opening	0.990 (0.982–0.995)	0.987 (0.977–0.994)	0.887 (0.793–0.943)	0.975 (0.955–0.987)
Height of left nostril opening	0.994 (0.989–0.997)	0.987 (0.976–9.993)	0.792 (0.620–0.894)	0.981 (0.964–0.990)
Columella	0.981 (0.965–0.990)	0.970 (0.945–0.985)	0.734 (0.517–0.864)	0.912 (0.832–0.956)

*ICC* intraclass correlation coefficient.

### Patients with cleft lip

In the conducted study at Siriraj Hospital, 15 patients presenting with cleft lip and associated nose deformities underwent corrective interventions. This cohort was constituted by nine cases of unilateral cleft lips and six bilateral. The distribution among the patients was nine females and six males, averaging an age of 8.7 ± 8.1 years. Notably, no discernible differences were observed in the measured parameters—namely height, width, and columella—based on either the side of the nostril or gender (Table 3).

**Table 3:** Gender-based distinction in nostril opening measurement of cleft patients.

	Female (n = 9)	Male (n = 6)	Total (n = 15)	p-value						
Age (year)	11.8 ± 8.9	4.1 ± 3.5	8.7 ± 8.1	0.01						

Parameter	Female (n = 9)			Male (n = 6)			Total (n = 15)			p-value
	Right	Left	Both	Right	Left	Both	Right	Left	Both	
Measurement of nostril opening										
Height (mm)	9.9 ± 1.1	9.7 ± 0.9	9.8 ± 1.0	9.9 ± 2.3	9.8 ± 1.5	9.9 ± 1.9	9.9 ± 1.6	9.8 ± 1.1	9.8 ± 1.4	0.055
Width (mm)	7.1 ± 0.6	7.4 ± 1.3	7.2 ± 1.0	6.4 ± 0.9	6.4 ± 1.6	6.4 ± 1.3	6.8 ± 0.8	7.0 ± 1.5	6.9 ± 1.2	0.937
Columella (mm)	5.9 ± 0.8			5.6 ± 1.0			5.8 ± 0.9			0.518

Direct measurements were juxtaposed with those derived from a program, revealing discrepancies in nostril widths of 0.7 mm for the right side and a mere 0.1 mm for the left. Height measurements exhibited a consistent difference of 1.1 mm for both nostrils. The columella measurements further presented a mean discrepancy of 1.1 mm (Fig. 3b).

Correlation analysis, employing the Bland–Altman plot, accentuated a strong concordance between the two methods of measurement across all parameters. With 95% of the observations landing within two standard deviations of the mean, the respective mean deviations for height, width, and columella stood at − 1.1 mm, − 0.3 mm, and − 1.1 mm (Fig. 4d–f) The intraclass correlation coefficients further underscored a commendable reliability, ranging from good to excellent for each parameter under consideration (Table 4).

**Table 4:** Intra- and inter-rater reliabilities of direct and program-based patients’ nostril opening measurement.

Parameters	Intra-rater reliability ICC (95% CI)		Inter-rater reliability ICC (95% CI)	
	Direct measurement	Program measurement	Direct measurement	Program measurement
Width of right nostril opening	0.955 (0.893–0.983)	0.977 (0.946–0.992)	0.768 (0.429–0.916)	0.919 (0.789–0.971)
Height of right nostril opening	0.988 (0.961–0.996)	0.977 (0.944–0.991)	0.944 (0.864–0.980)	0.923 (0.813–0.972)
With of left nostril opening	0.979 (0.951–9.992)	0.955 (0.881–0.984)	0.943 (0.867–0.979)	0.797 (0.532–0.925)
Height of left nostril opening	0.964 (0.905–0.987)	0.978 (0.948–0.992)	0.918 (0.809–0.970)	0.970 (0.929–0.989)
Columella	0.970 (0.929–0.989)	0.948 (0.878–0.981)	0.799 (0.530–0.926)	0.966 (0.904–0.988)

*ICC* intraclass correlation coefficient.

## Discussion

Rectifying abnormalities in cleft patients necessitates a range of surgical procedures to achieve satisfactory functional and esthetic outcomes. Beyond mastering the surgical technique, a key challenge is maintaining the stability of the nasal correction. Many studies highlighted the occurrence of postoperative cartilage collapse. Specifically, 20–35% of cleft lip nasal deformity patients have been reported to require a second correction of their deformities^13,14^. This challenge is particularly significant in the Asian population, which often has distinct nasal characteristics such as thick sebaceous skin over the nasal tip and supratip area, weak lateral cartilage, retracted columella, and thick alar lobules^15,16^.

Nostril retainers are integral in the postoperative phase, serving primarily to prevent nasal ala collapse and retain the nasal contour. Their effectiveness has been well-established over many decades^1,7,17^. However, there are inherent challenges. One notable drawback is the need to frequently adjust the retainer to match the patient's growth, with adjustments needed every 2–4 weeks^18,19^.

Nakajima et al.^1^ designed a nostril splint with the intent of maintaining nasal overcorrection and being easily adaptable to individual nostril contours. They used quick set moldable silicone rubber for the nostril post-surgery, which was fixed to the nasal dorsum by suturing and left for two weeks. These retainers were remolded according to changes in the patient's contour at outpatient clinics over 3–4 months. A notable concern from parents was the difficulty in cleaning these nostril splints.

In Thailand, the cost associated with a commercial nostril retainer can range between 168.59 to 2697.43 USD, especially if there is a need to alter the nostril splint every two weeks for a six-month duration.

In Russia, there were limitations regarding the availability of nostril retainers. Recognizing the importance of these retainers for cleft patients, Khonsari et al.^13^ collaborated with orthotists to fashion nostril retainers from readily available medical equipment like endotracheal tubes. To avoid the need for adhesive, they later designed a non-adhesive nostril retainer using 3D images from the patient's computed tomography scan.

Several studies^8–10^ have leveraged image analysis for cleft patients, either using 2D or 3D images to analyze facial surface asymmetry and predict post-operative outcomes. Such analysis provides both visual and quantitative insights into cleft deformities and significantly aids reconstructive planning, thereby improving surgical outcomes.

Our study took a different approach, utilizing 2D photographic parameters to generate a patient-specific 3D model for nostril retainers. Measurements taken directly from thirty Thai volunteers showed the average height and width of the nostril opening to be 12.5 ± 1.3 mm and 8.6 ± 1.4 mm, respectively, in a resting position (Table 1). Excessive postoperative distension could lead to secondary rhinoplasty due to the widening of the nostril opening. Thus, the correct size of a postoperative nostril retainer is crucial for a successful treatment outcome^20^.

Our process leveraged common devices. All participants were photographed from specific angles, ensuring the angle was parallel to a green sticker. 2D photographs from mobile phones were then imported into a program that marked the nasal opening area. This program, based on the nostril opening's outline, generated a nostril retainer and allowed separate designs for each side of the opening. The program then calculated the nostril opening's width and height by converting pixel numbers into millimeters, using green stickers of 0.3 × 0.3 cm and 0.5 × 0.5 cm for reference.

The gap between direct measurements and program measurements in healthy volunteers was less than 2 mm (Fig. 3a), while in cleft patients, the inaccuracy hovered around 1 mm (Fig. 3b). The nasal aperture estimated by a program is somewhat larger than that measured directly in volunteers or patients. This inaccuracy could be influenced by the photograph's angle and the presence of a green mark on patients.

With cooperation from one the patient's family, an image of the patient was sent to our team and a 3D model nostril retainer developed by our program was printed out and returned to the patient's home. Compared to a commercial retainer, which costs 77.2 USD and requires patients to try on a sizer at the hospital, this production procedure and shipment costs only 11.7 USD. Our service help patients and their families personally manage the shape of the patient's nasal aperture. In addition, the patient's family can save money on treatment by modifying the nostril retainer more frequently to fit the postoperative shape of the nares. Specifically in columella width, which may compromise the fit and direction of each nostril retainer crus.

In situations like the COVID-19 pandemic, our custom-made 3D nostril retainers could offer significant advantages by facilitating remote follow-ups. By using 2D photography and 3D modeling software, we can enable remote consultations, thus reducing in-person appointments and enhancing accessibility for patients in remote locations.

However, our study has limitations, including a small sample size. Future research should aim for larger sample sizes, explore other measuring techniques, and consider more advanced software to improve the customization and fit of nostril retainers, especially those with user-friendly features.

## Conclusions

Two-dimensional photographs of cleft lip and cleft lip nose deformities can be used to create a custom-made, three-dimensional nostril retainer that can maintain the patient's nare postoperatively without visiting hospital, thereby reducing the cost of care and increasing patient and family satisfaction.

### Supplementary Information

Supplementary Figure 1.

## Data Availability

The datasets used and/or analysed during the current study available from the corresponding author on reasonable request.
