# Author Correction: Utilization of images and three-dimensional custom-made nostril retainer fabricate for patients with cleft lip and cleft lip nose deformities at Siriraj Hospital: preliminary phase

**DOI:** 10.1038/s41598-024-54022-y

**Published:** 2024-02-13

**Authors:** Irin Chaikangwan, Nutcha Yodrabum, Worapan Kusakunniran, Rachata Tachavijijaru, Chongdee Aojanepong

**Affiliations:** 1 Division of Plastic and Reconstructive Surgery, Department of Surgery, Faculty of Medicine Siriraj Hospital, Mahidol University, Bangkok, 10700 Thailand; 2 Faculty of Information and Communication Technology, Mahidol University, Nakhon Pathom, Thailand

Correction to: *Scientific Reports* 10.1038/s41598-023-46327-1, published online 04 November 2023

The original version of this Article contained an error in the protocol number.

In the Methods section, under the subheading ‘Study subjects’,

“Institutional approval was secured from Siriraj Hospital's Institutional Review Board, as evident from protocol number 174/2564 (IRB4).”

now reads:

“Institutional approval was secured from Siriraj Hospital's Institutional Review Board, as evident from protocol number 174/2564 (IRB4) (COA no. Si 536/2021).”

The original Article has been corrected.

