# Supplementary material for: Utilization of images and three-dimensional custom-made nostril retainer fabricate for patients with cleft lip and cleft lip nose deformities at Siriraj Hospital: preliminary phase
Source: Sci Rep. 2023 Nov 4;13:19109. doi: 10.1038/s41598-023-46327-1 (PMC10625571; doi:10.1038/s41598-023-46327-1)

**Figure legends**

**Supplementary figure 1** Step-by-step illustration of setting up the basilar view to import the best quality image into the application.


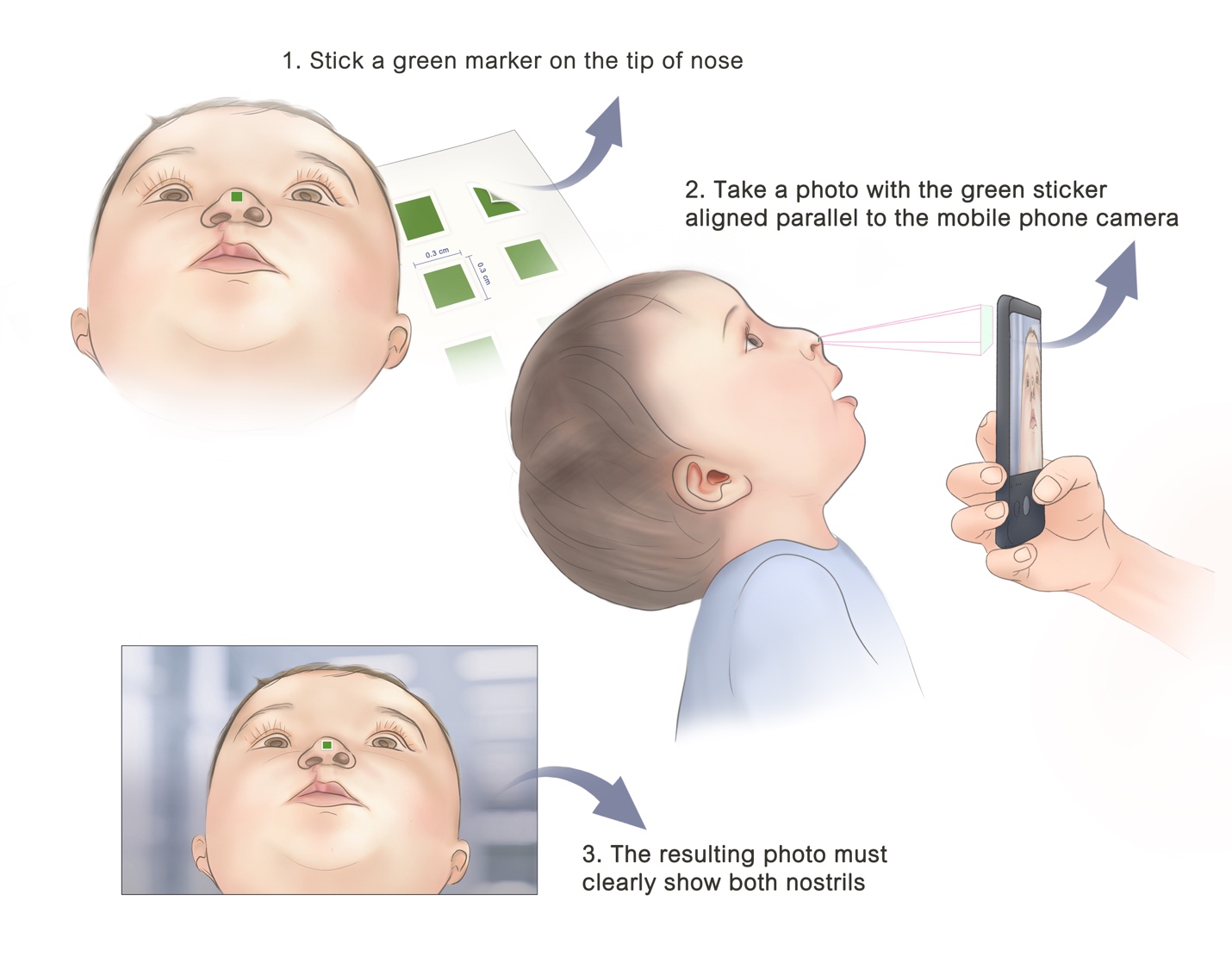

Supplement: Supplementary file 1 — Supplementary Figure 1. [file 41598_2023_46327_MOESM1_ESM.docx]
